# Variations in climatic suitability and planting regionalization for potato in northern China under climate change

**DOI:** 10.1371/journal.pone.0203538

**Published:** 2018-09-27

**Authors:** Junfang Zhao, Xin Zhan, Yueqing Jiang, Jingwen Xu

**Affiliations:** 1 State Key Laboratory of Severe Weather, Chinese Academy of Meteorological Sciences, Beijing, China; 2 College of Resources, Sichuan Agricultural University, Chengdu, China; 3 National Meteorological Center, Beijing, China; Fred Hutchinson Cancer Research Center, UNITED STATES

## Abstract

Investigating the variations in crop climatic suitability and planting regionalization can provide scientific evidence for ensuring food security under climate change. In this study, variations in climatic suitability and planting regionalization for the potato in northern China were investigated based on daily data from 1965 to 2014 collected at 321 agro-meteorological observation stations located throughout the region. Northern China was divided into three areas, including Northwest China, North China and Northeast China. The agricultural climatic suitability theory and the fuzzy mathematics method were applied. The potato growth seasons were divided into threestages:from sowing to emergence, from emergence to flowering and from flowering to maturity. The comprehensive climatic suitabilityindex (C), which varied from 0 to 1, was established to evaluate the effects of climate change on potato planting. The results showed that, from 1965 to 2014, the C value in the study area increased 0.002 every ten years over the past 50 years with an average of 0.706, benefitting potato growth in the vast area of northern China. Nonetheless, precipitation was found to be the main climatic factor restricting potato growth in northern China. For spatial distribution, the C value showed a gradually declining trend from east to west, decreasing westward and southward over the past 50 years. For the growth season, the C value varied during different potato growth stages over the past 50 years. The C value increased during the sowing-emergence stage and decreased during the emergence-flowering stage and the flowering-maturity stage. The decreased C during the later growth stages would directly affect the quality and yield of the potato, mainly because the flowering-maturity stage was associated with potato tuber enlargement and starch accumulation. Variations in potato planting regionalization in northern China over the past 50 years were evident. Climate change was more beneficial to potato cultivation in northeast China where the highly suitable areas had clearly expanded. However, potato cultivation was most negatively affected in northwest China where the middle suitable areas had receded. Our findings have important implications for improving climate change impact studies and agricultural production to cope with ongoing climate change.

## Introduction

Climate change has major impacts on crop suitability and yield variability in agricultural systems through shifts inweather patterns (e.g., precipitation and temperature) and extreme weather events (e.g., floods, droughts and storms) [1,2,3]. Rising temperatures may detrimentally affect crop production, causing shortened growing seasons and reduced time for biomass accumulation, except in the coolest regions where temperatures are currently below the optimum range [4]. Contrarily, elevated atmospheric CO_2_ concentrationmay increase production for some crops [5]. These changes are expected to continue and may even accelerate in the future, resulting in potentially severe but highly uncertain impacts on agricultural land systems [6]. The increased adverse impacts of climate change on agriculture have caused inter-national communities, experts, policy-makers, and farmers to assess the suitability of the climate for agriculture in a given area, then propose and apply adaptation strategies to improve or perhaps maintain agriculture in some regions [7,8,9,10].

The potato (*Solanumtuberosum L*.) is the fourth-most important food crop consumed worldwide after wheat (*Triticumaestivum L*.), rice (*Oryza sativa L*.) and corn (*ZeamaysL*.) [11]. Currently, there are approximately 5 million hectares of potato cropland in China, accounting for more than 20% of the total global potato acreage [12]. Total potato production in China is nearly 90 million tons, accounting for 25% of global potato production [13]. Northern China is the largest potato-producing region in China, with approximately 49% of the country’s planting area. This region has become China’s primary producer of seed potatoes and processed potatoes. Potato is sensitive to changes in major environmental factors [14], and its yield is influenced by light, temperature and water [15]. However, northern China is hindered by relatively fragile ecosystems and poor agricultural technology, making it sensitive to climate change and directly affecting potato yield and quality. Therefore, identifying the characteristics of potato climatic suitability and the variations in planting regionalization for potato in northern China is an effective approach to climate change adaptation and provides an opportunity to ensure regional food security.

Evaluation models that describe crop growth and development over time as a function of environmental factors can be used to estimate the effects of climate change on crop suitability [8,12,16,17]. For example, Caubel et al [8] developed an assessment method based on agro-climatic indicators calculated over phenological periods (eco-climatic indicators) for crop climate suitability. He et al [12] used a temperature thermal response coefficient model to calculate a temperature suitability value for potato phenology, conducting precise temporal and spatial evaluations on temperature suitability for potato growth in China. Nguyen et al [16] developed a GIS-based multi-criteria analysis procedure to evaluatethe suitability of temperature and precipitation for the winter wheat and summer maize cropping systems in the Huang-Huai-Hai Plain, China. In China, the impacts of climate change on potato over the past few decades have been a major cause of concern [18,19,20,21]. Wang [18] found global climate warming had caused adverse effects on potato yield in semi-arid regions of northwest China from 1961 to 2010 based on WOFOST crop model. Zhao et al [21] adopted the first difference method to disentangle the contributions of climate change to potato yield in northern China. They concluded that the potato yield in this area was the most sensitive to variation of the diurnal temperature range followed by radiation, precipitation and reference crop evapotranspiration. However, the above research mainly focuses on the relationship between climate change and potato yield at a small scale. To date, the impacts of climate change on potato suitability and planting regionalization in northern China at a regional scale have not been thoroughly evaluated. Understanding how different climate factors interact and impact potato suitability and regionalization is essential when making decisions on how to adapt to the effects of climate change.

The objectives of the present study are to: (1) understand characteristics of temperature suitability, precipitation suitability and light suitability in northern China during each potato growth stage using robust observational evidence based on the agricultural climatic suitability theory and the fuzzy mathematics method; (2) explore the effects of climatic variables on comprehensive potato suitability using long-term datasets; (3) quantitatively evaluate the variations in planting regionalization of potato in northern China from 1965 to 2014. These findings are significant for substantially improving our understanding of crop suitability in response to climate change on a regional scale in China.

## Materials and methods

### Study area

**Fig 1:**
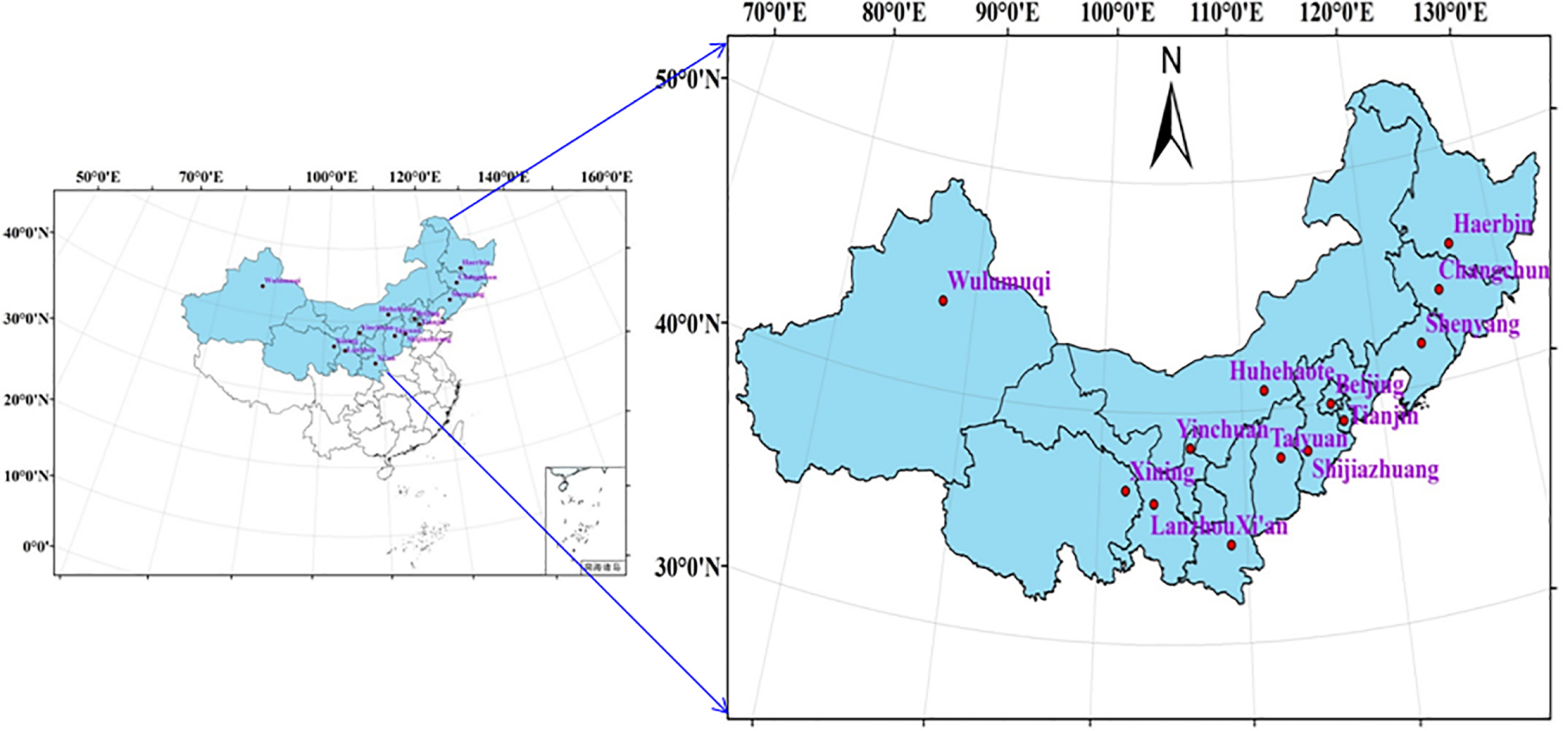
Study area location innorthern China.

Northern China is located at longitude 73–136°E and latitude 31–54°N, with a total area of approximately 499.5×10^4^ km^2^. Northern China is divided into three areas (Northwest China, North China and Northeast China) and includes the following 13 provinces: Shaanxi, Gansu, Qinghai, Ningxia, Xinjiang, Beijing, Tianjin, Hebei, Shanxi, Inner Mongolia, Heilongjiang, Jilin and Liaoning (Fig 1). Affected by many local factors such as topography, altitude, etc, the climate in this area is arid, semi-arid and sub-humid. In general, this region is characterized by strong winds, long periods of sunshine and solar radiation. Vegetation types include forests, meadows, grassland, steppes, scrubs, desert and cultivated vegetation. Northern China is a particularly important area for potato cultivation in the country as a whole. Typically, local potatoes are planted in spring (mid to late April—early May) and harvested in autumn (September), with only one planting season per year. Potato yield in this region accounted for 38.3% of China’s total potato yield from 2004 to 2012 [21]. As such, potatoes play an important role in the development of this region.

### Data

Daily climate variables including maximal and minimal air temperature, average air temperature, precipitation, solar radiation, relative humidity, and wind speed, were obtained from 321 agricultural meteorological stations in northern China between 1965 and 2014. Information on climatic variables was provided by the National Meteorological Information Centre.

Long-term datasets for potato growth and development were collected from the National Bureau of Statistics and the agricultural meteorological stations in northern China between 1965 and 2014. These data were mainly used to calculate the temperature suitability of potato and the precipitation suitability of potato.

**Fig 2:**
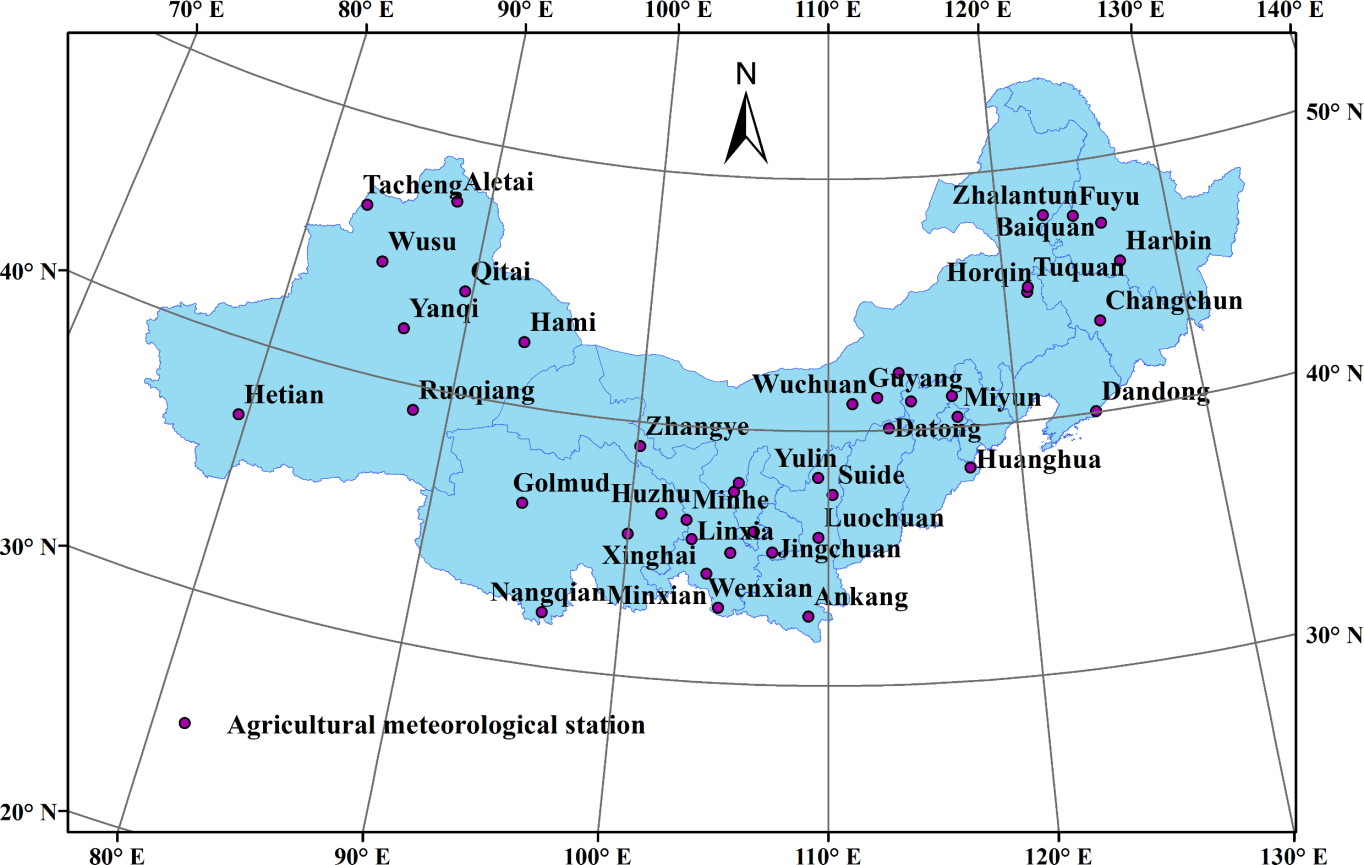
Distribution of agricultural meteorological stations selected in the study area.

To evaluate the climatic suitability of potato, 43 agricultural meteorological stations were selected in the study area based on the unique geographical and climatic characteristics of northern China (Fig 2). The selection principles were as follows: (1) the selected station was located in the potato-producing area of northern China; (2) several stations selected in each province represented the local climate and the geography; (3) each representative station had geographical and climatic differences and relative independence from one another.

### Climatic suitability evaluation of potato in northern China

The relationship and the degree of coordination between climate and crops often need to be described in a quantitative form. An evaluation model of crop climatic suitability can be developed for the growth of crops based on crop climatic resources, revealing the intrinsic link between climate and some crops. This can further explain the benefits and restrictions to crop growth and regional distribution in different climates, providing an important theoretical basis for the improvement of technology, management and allocation of agricultural production resources. In this study, the agricultural climatic suitability theory [22] and the fuzzy mathematics method [23] were applied. Potato growth season in northern China was divided into 3 stages: from sowing to emergence, from emergence to flowering and from flowering to maturity. The climatic suitability evaluation of the potato included four parts: temperature suitability, precipitation suitability, light suitability and comprehensive climatic suitability.

#### Brief introduction of agricultural climatic suitability theory

Generally speaking, the relationship between crop and climate is mainly reflected by the response of crops to climate and the suitability of crops to climate. Suitability not only reflects the anastomosis of crops in a certain climate, including growth and development, yield level and distribution, but also shows the advantages and disadvantages and degree of resources provided by the environment for crops. Light, heat and water resources are the three most basic elements of agricultural climatic resources. They provide crops with essential materials and energy for growth. All the factors of agricultural climatic resources have a certain suitable range for the growth of crops. The method of fuzzy mathematics can be used to define the suitability of agricultural climatic resources for the growth and development of crops to [0 and 1] interval. 0 of them indicates that the climate is completely unsuitable for the growth and development of crops, and 1 indicates that the climate is the most suitable for the growth and development of crops. The other is a continuous transition state [22].

#### Fuzzy mathematics method

Fuzzy mathematics forms a branch of mathematics related to fuzzy set theory and fuzzy logic. It started in 1965 after the publication of Lotfi Asker Zadeh's seminal work Fuzzy sets [23]. A fuzzy set is a class of objects with a continuum of grades of membership. Such a set is characterized by a membership (characteristic) function which assigns to a each object a grade of membership ranging between zero and one [24].

#### Temperature suitability of potato

In order to describe the temperature suitability of potato, the temperature suitability index was adapted. According to observed conditions of potato growth and development in northern China, the formula for the temperature suitability index could be expressed as [22]:

$$S(T)={\frac{\left[(T-T_{1}){\left(T_{2}-T\right)}^{B}\right]}{\left[(T_{0}-T_{1}){\left(T_{2}-T_{0}\right)}^{B}\right]}}_{}$$ (1)

$$B=\frac{T_{2}-T_{0}}{T_{0}-T_{1}}$$ (2)

where *S(T)* was the temperature suitability index of potato; *T*_*1*,_*T*_*0*_ and *T*_*2*_ were the lower limit of growth temperature, the most suitable growth temperature and the upper limit of growth temperature for potato, respectively; *T* was the average temperature during the potato growth season; *B* was a constant. The lower limit of growth temperature, the most suitable growth temperature and the upper limit of growth temperature for the potato were listed in Table 1 [25,26].

**Table 1:** Temperature parameters of potato during the different growth stages in northern China.

Variable	Growth stage		
	From sowing to emergence	From emergence to flowering	From flowering to maturity
T_1_	5	7	8
T_0_	16	19	17
T_2_	25	30	29
B	0.82	0.92	1.33

Notes:*T*_*1*,_*T*_*0*_ and *T*_*2*_ were the lower limit of growth temperature, the most suitable growth temperature and the upper limit of growth temperature, respectively; B was constant.

#### Precipitation suitabilityof potato

The precipitation suitability index was used to evaluate the precipitation suitability of potato in Northern China. Itwas expressed as follows [7]:

$$\mathrm{When}R<ET_{m},S(R)=R/ET_{m}$$ (3)

$$\mathrm{When}R\geq ET_{m},S(R)=ET_{m}/R$$ (4)

where *S(R)* was the precipitation suitability index of potato; *R* was the precipitation in the specific growth period of potato; *ET*_*m*_ was the physiological water requirement of potato during the potato growth season and defined as *K*_*c*_×*ET*_*0*_, where *ET*_*0*_ was the reference evapotranspiration calculated using the FAO Penman–Monteith method (FAO) [7,27] with *K*_*c*_ as crop coefficient that depended on the growth stage (Table 2) [28].

**Table 2:** Crop coefficientof potato during the different growth stages in northern China.

Variable	Growth stage		
	From sowing to emergence	From emergence to flowering	From flowering to maturity
Kc	0.4	1.15	0.75

#### Light suitability of potato

The light suitability index was used to evaluate the light suitability of potato in Northern China in this study. Potatoes are heliophiles. Its growth morphogenesis and yield have strong reactions dependent on light intensity and sunshine. Light affects not only potato growth but also assimilation distribution [21]. It was generally believed that when the sunshine hours exceeded 70% of the duration possible, the available light would be suitable for potato growth; that is, the light suitability of the potato was 1. Therefore, the formula for the light suitability index of potato in northern China was expressed as [7]:

$$\mathrm{When}S<S_{0},S(S)=l^{-{\left[(S-S_{0})/b\right]}^{2}}$$ (5)

$$\mathrm{When}S\geq S_{0},S(S)=1$$ (6)

where *S(S)* was the light suitability index of potato; *S* was the actual sunshine time; *S*_*0*_ was total sunshine hours when in excess of 70% of the duration possible; b was a constant that could be determined by fitting.

#### Comprehensive climatic suitability for potato during the whole growth season

The growth, development and yield of the potato are affected by environmental factors, especially the combined effects of temperature, precipitation and light. The following comprehensive climate suitability index was established for evaluating the light suitability of potato in Northern China:

$$C=\sum_{i=1}^{n}\left(w_{1}S(T{)}_{i}+w_{2}S(R{)}_{i}+w_{3}S(S{)}_{i}\right)$$ (7)

where *C* was the comprehensive climatic suitability index of the potato during the whole growth season; *w*1 was the proportion of the average correlation coefficient between temperature suitability index and per unit potato yield in each growth stage, and the correlation coefficient sum between all climatic factors suitability index and per unit potato yield; *w*2 was the proportion of the average correlation coefficient between precipitation suitability index and per unit potato yield in each growth stage, and the correlation coefficient sum between all climatic factors suitability index and per unit potato yield; *w*3 was the proportion of the average correlation coefficient between light suitability index and per unit potato yield in each growth stage, and the correlation coefficient sum between all climatic factors suitability index and per unit potato yield; *i* was the growth stage; *n* was the number of growth stages involved in the entire growth process; *S(T)* was the temperature suitability index of potato; *S(R)* was the precipitation suitability index of the potato; *S(S)* was the light suitability index of the potato.

In this study, the weight *w* was determined by the correlation coefficient method. The process of determining the weight using the correlation coefficient method was as follows: first, the correlation coefficient matrix among temperature, precipitation or light and per unit potato yield for given growth stages (from sowing to emergence, from emergence to flowering and from flowering to maturity) from 1965 to 2014 was determined; second, the average correlation coefficient between a main climatic factor (temperature, precipitation or light) suitability index and per unit potato yield during the whole growth stage was calculated; finally, the weight values were determined according to the proportion of average correlation coefficient between a climatic factor suitability index and per unit potato yield during the whole growth stage, and the correlation coefficient sum between all climatic factors suitability index and per unit potato yield. Key climate factor weights during the different potato growing stage in northern China were presented in Table 3.

**Table 3:** Key climate factors weights during the potato growing season in northern China.

Weight value	Major climatic factors		
	Temperature	Precipitation	Light
w	0.37	0.30	0.33

### Climatic regionalization of potato planting in northern China

Based on previous research [18,29,30], the climatic regionalization of potato planting in northern China was divided into four parts in this study: high suitability region (IV), middle suitability region (III), general suitability region (II) and lowsuitability region (I) (Table 4).

**Table 4:** Climatic regionalization of potato planting in northern China.

C value	Comprehensive climate suitability	Climatic regionalization level for potato planting
0.80–1.00	High suitability	Ⅳ
0.65–0.80	Middle suitability	Ⅲ
0.55–0.65	General suitability	Ⅱ
0.00–0.55	Low suitability	Ⅰ

### Impact of climate change on potato planting regionalization in northern China

In the analysis of the variations in potato planting regionalization in northern China from 1965 to 2014, two time periods were considered: the first 25 years (1965–1989) and the last 25 years (1990–2014).Variations in the four climatic regionalization areas (high, middle, general and low suitability regions) for potato planting in northern China were compared between the first 25 years and the last 25 years.

## Results

### Climate change characteristics during potato growth season in northern China

Average temperature in the study area increased significantly from 17.52 in 1965 to 18.51 in 2014. The warming periods were mainly concentrated in 1970s and 1990s. As for the distribution of average temperature, it was higher in the East and the Northwest, gradually decreasing in the southwestern region of Qinghai province and the central region of Inner Mongolia plateau. Annual precipitation in the study area decreased during the potato growth season and fluctuated greatly in the interannual range. The spatial distribution of annual precipitation was high in the Southeast and low in the Northwest, decreasing from coast to inland. Sunshine hours from 1965 to 2014 in this area decreased from 8.67 hours in 1965 to 7.33 hours in 2014. Moreover, the obvious declines were found in 1960s and 1970s. The spatial distribution of sunshine hours was high in the Northwest and low in the Southeast, decreasing from the Northwest to the Southeast.

### Temperature suitability characteristics during potato growth season in northern China

The average temperature suitability index during the whole growth season from 1965 to 2014 in northern China showed a slightly decreasing trend, with an average of 0.873. The maximum average temperature suitability index value was found in 1998 (0.921), and the minimum value was found in 2004 (0.704).

**Fig 3:**
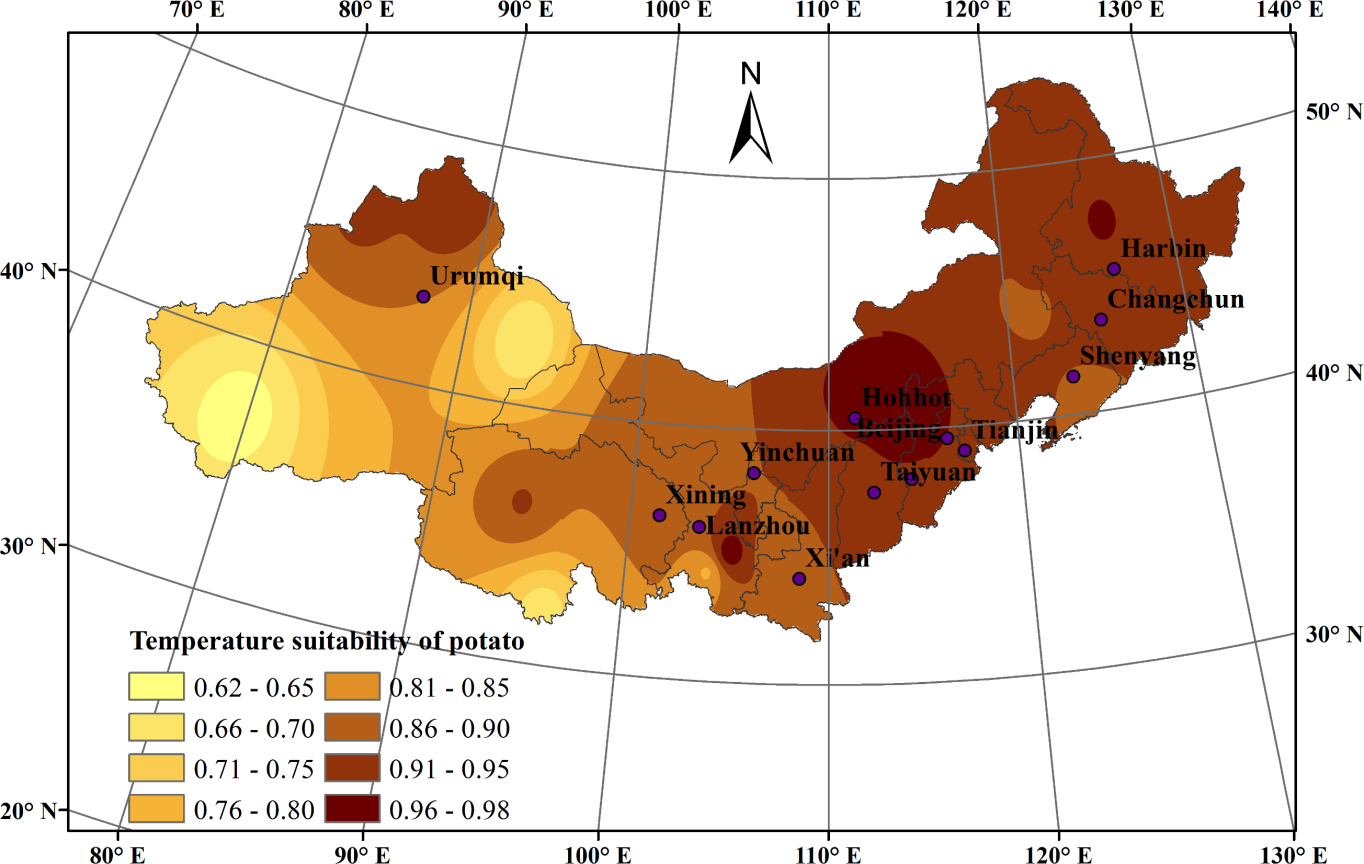
Temperature suitability distribution during the potato growth season in northern China during 1965–2014.

In general, the spatial distribution of the average temperature suitability index of potato during the whole growth season for 1965–2014 showed a gradually declining trend from the eastern region to the western region (Fig 3). The areas with the highest values of temperature suitability were mainly concentrated in Baiquan (0.955) in Heilongjiang, Changchun (0.910) in Jilin, Wuchuan (0.970) and Tuquan (0.940) in Inner Mongolia, Datong (0.950) in Shanxi, Zhangbei (0.907) in Hebei, Tongwei (0.982), Tacheng (0.916) and Aletai (0.945) in northwestern Xinjiang. Conversely, the temperature suitability index was low in most areas of Qinghai and Xinjiang, particularly in NangQian (0.672) and Hetian (0.624), due to the large daily temperature range.

As far as the growth stage of potato was concerned, the average temperature suitability varied during the different potato growth stages from 1965 to 2014. During the sowing-emergence stage, the average temperature suitability index increased slightly, fluctuating between 0.886 and 0.901. During the emergence-flowering stage, the average temperature suitability index decreased from 0.937 to 0.909, but maintained a relatively high value. During the flowering-maturity stage, the average temperature suitability index was mostly stable between 0.800 and 0.805. In summary, the temperature in most areas of northern China could effectively meet the demands of potato growth, and was suitable over the past 50 years.

### Precipitation suitability characteristics during potato growth season in northern China

During the potato growing seasons (April-September) from 1965 to 2014 in northern China, the average precipitation suitability index during the whole growth season showed a slightly decreasing trend and declined 0.008 every ten years, with an average of 0.452. The maximum precipitation suitability index value was found in 2004 (0.816), and the minimum value was found in 2012 (0.307).

**Fig 4:**
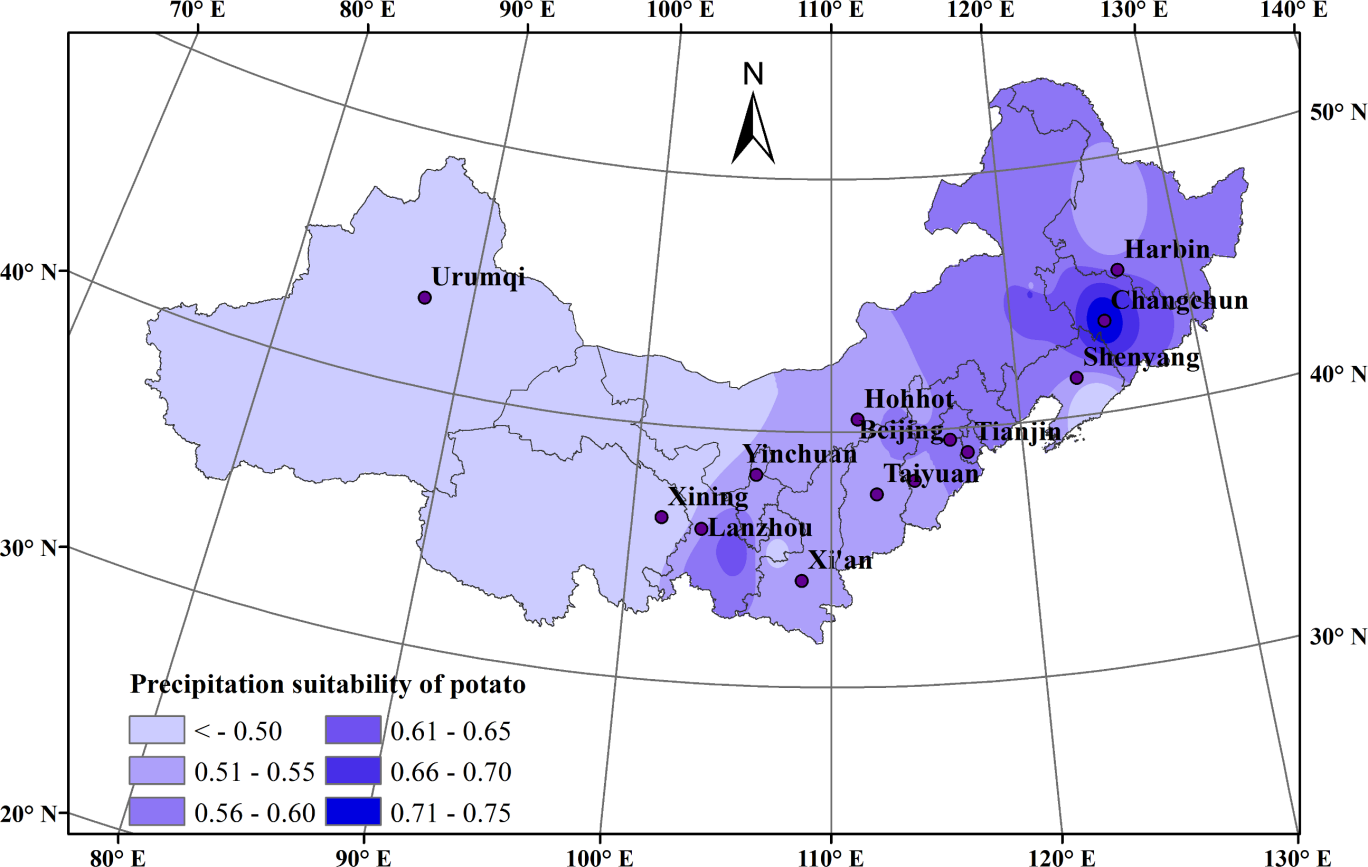
Precipitation suitability distribution during the potato growth season in northern China during 1965–2014.

The spatial distribution of average precipitation suitability index of the potato during the whole growth season showed a gradually declining trend from east to west (Fig 4). The areas with the highest values of precipitation suitability were mainly concentrated in Changchun (0.746) in Jinlin, Horqin (0.687) in Inner Mongolia and Tongwei (0.645) in Gansu. The precipitation suitability index was approximately 0.500 in some regions including Shanxi, Shaanxi, western Inner Mongolia, and Liaoning, all of which were found in the generally suitable region. Conversely, most areas of Xinjiang and Qinghai showed low precipitation suitability, including Hami (0.044), Golmud (0.115), and Hotan (0.262).

Average precipitation suitability index values decreased during each growth stage from 1965 to 2014. During the sowing-emergence stage, the average precipitation suitability index decreased slightly from 0.348 to 0.346. During the emergence-flowering stage, the average precipitation suitability index decreased from 0.445 to 0.440. During the flowering-maturity stage, the change in average precipitation suitability index was relatively large and decreased 0.07 every ten years, fluctuating between 0.573 and 0.540.

In summary, these results showed that the precipitation suitability of potato in most areas was generally low. Over the past 50 years, the precipitation suitability index of potato in this area overall decreased. Moreover, the precipitation suitability index of potato during each growth period displayed a downward trend, especially during the flowering-maturity stage. Because the flowering-maturity stage was related to the further development and tuber expansion of the potato, this downward trend would have direct adverse effects on potato yield. Based on these results, precipitation remained the main climatic factor restricting potato growth in northern China.

### Light suitability characteristics during potato growth season in northern China

With little precipitation and long periods of sunshine, northern China had a high light suitability index for potato growth over the past 50 years, with an average of 0.750. During the potato growing seasons (April-September) from 1965 to 2014, the average light suitability index of the potato showed an increasing trend, increasing 0.014 every ten years.The maximum value of light suitability index was found in 2013 (0.891), and the minimum value was found in 1995 (0.679).

**Fig 5:**
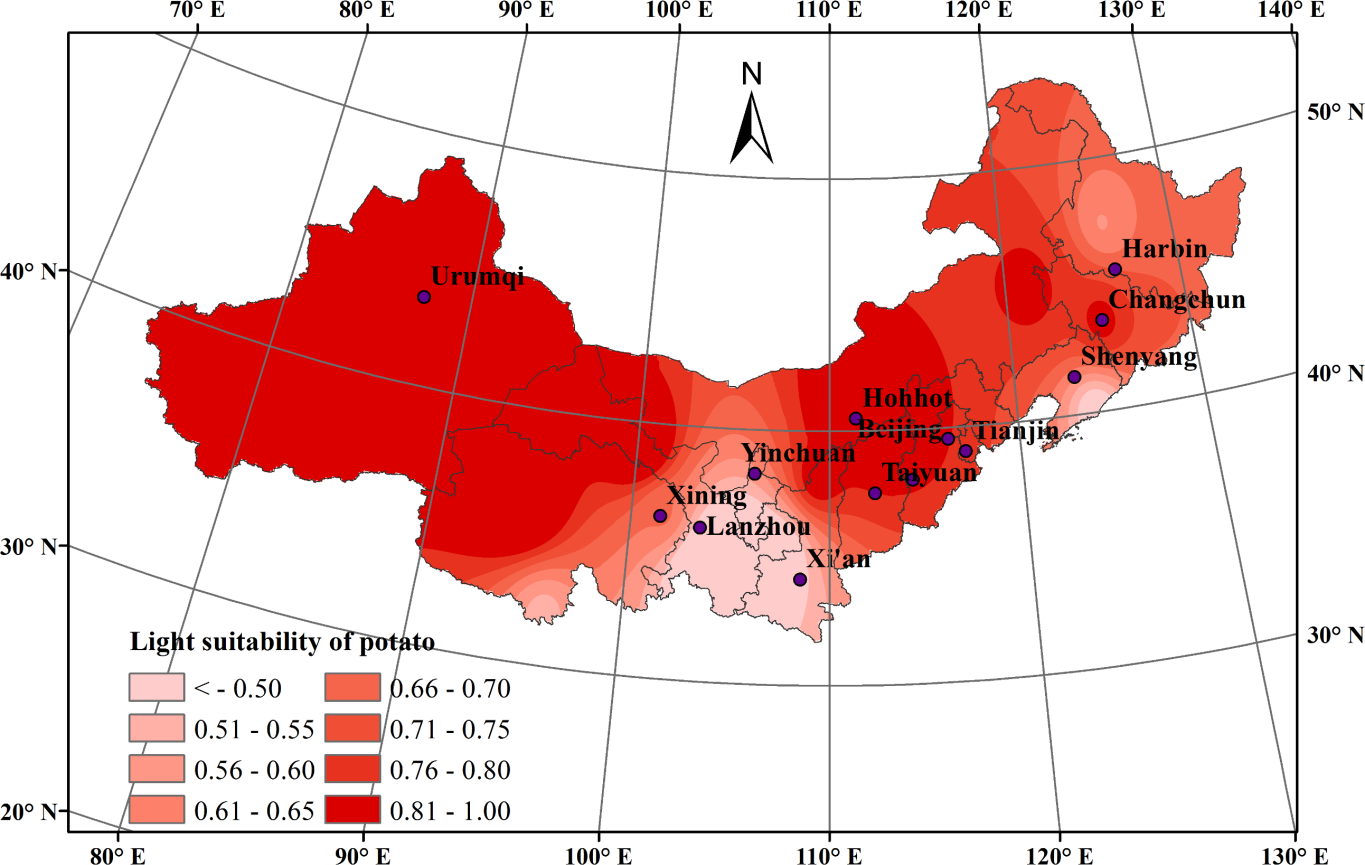
Light suitability distribution during the potato growth season in northern China during 1965–2014.

The spatial distribution of average light suitability index of the potato during the whole growth season showed a pattern of high values in the north and low values in the south, as well as high values in the western inland region and low values in the eastern monsoon region (Fig 5). Areas with high light suitability were mainly concentrated in Xinjiang, Qinghai, and central and western Inner Mongolia. Perennial light time was more than 70% of the illumination time, especially in some areas such as Aletai (1.000), Hami (1.000), Tacheng (1.000) in Xinjiang, Wuchuan (0.968) in Inner Mongolia, and Zhangye (0.971) in Gansu. The low light suitability areas were mainly concentrated in Gansu, southern Qinghai and northeast China, including Tongwei (0.241), Jingchuan (0.383), and Dandong (0.442). These areas were characterized by more clouds, more precipitation and less light. The light suitability index in other area was almost 0.800, which was suitable for potato growth.

The average light suitability index of potato increased over each growth stage. Over the past 50 years, the average light suitability index during the sowing-emergence stage increased from 0.757 to 0.821. During the emergence-flowering stage and the flowering-maturity stage, the average light suitability index increased from 0.790 to 0.805 and from 0.650 to 0.676, respectively. These results showed that light suitability in most areas of northern China was high overall and could meet the light demands of the potato, which was beneficial to potato cultivation, development and production. Moreover, the geographic range of the suitable planting region expanded over the past 50 years.

### Comprehensive climatic suitability characteristics during potato growth season in northern China

**Fig 6:**
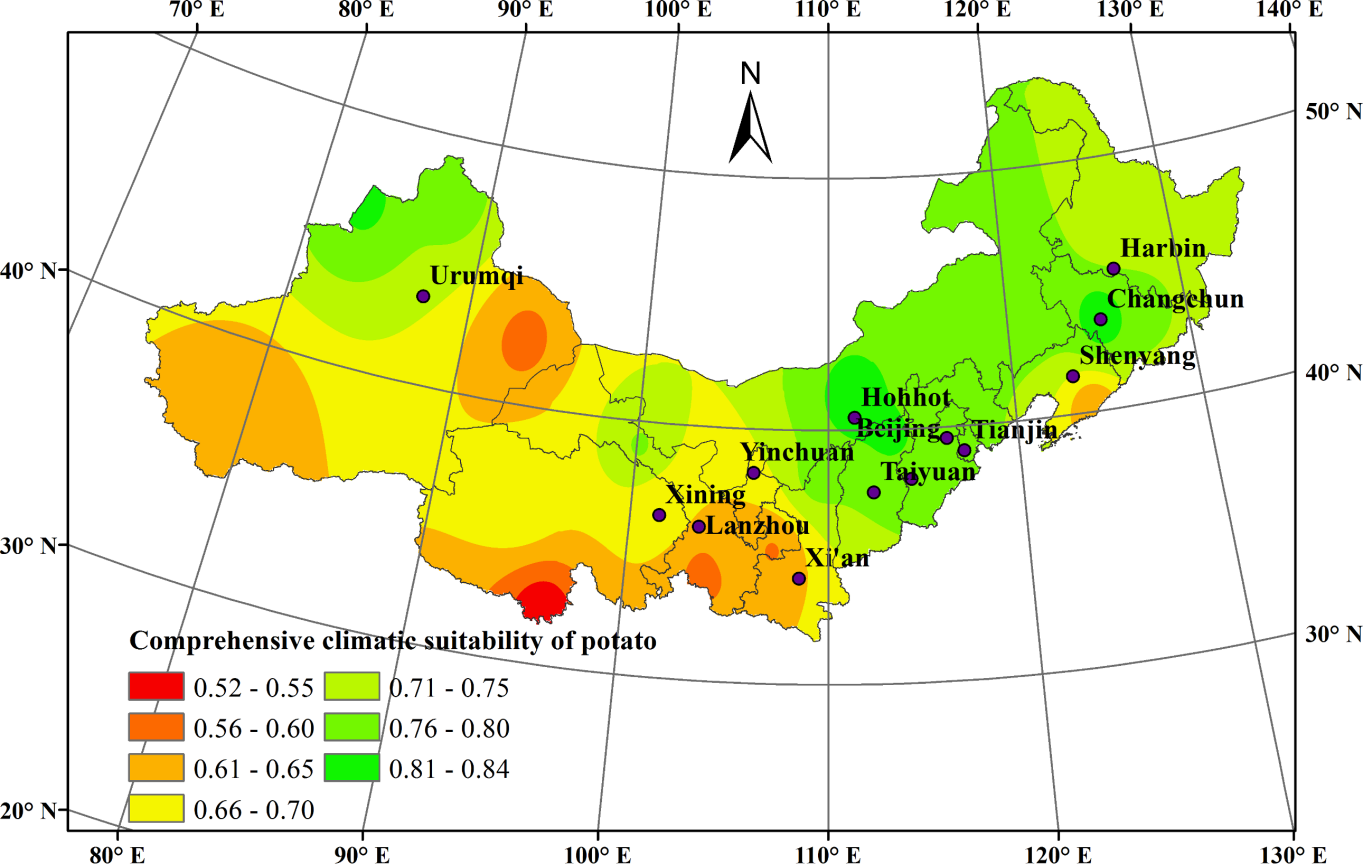
Comprehensive climatic suitability distribution during the potato growth season in northern China during 1965–2014.

The comprehensive climatic suitability index (C) of potato in the study area was high overall, increasing 0.002 every ten years over the past 50 years, with an average of 0.706. The spatial distribution oftheCshowed a pattern ofhigh values in the east and low values in the west (Fig 6).

The C value varied across different growth stages. Over the past 50 years, the average C value during the sowing-emergence stage increased from 0.886 to 0.901. However, during the emergence-flowering stage and the flowering-maturity stage, the average C value decreased from 0.937 to 0.909 and from 0.805 to 0.800, respectively. Overall, the C value over the past 50 years showed a slightly increasing trend during the whole growth season, benefitting potato growth in northern China.

### Planting regionalization for potato in northern China

**Fig 7:**
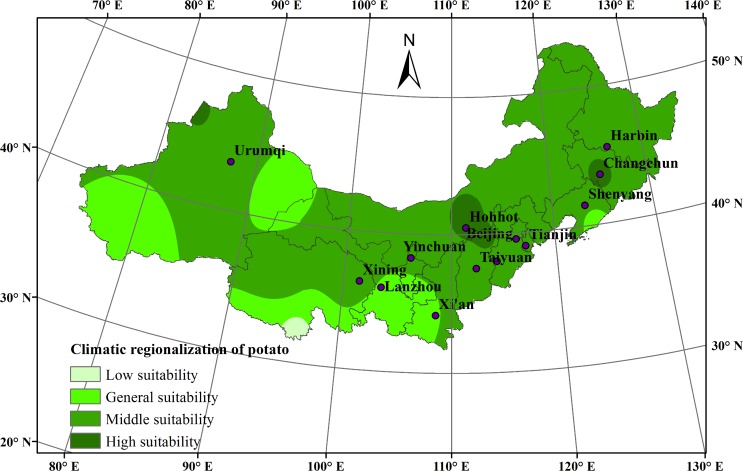
Average climatic regionalization of potato planting in northern China during 1965–2014.

Average climatic regionalization of potato planting in northern China was shown in Fig 7. The highly suitable areas were mainly distributed in central Inner Mongolia, northwestern Hebei and the Sanjiang Plain in northeast China. The average temperature during the potato growth season in this region was 18°C-20°C. Annual precipitation was approximately 400 mm-600 mm. These areas were cool, humid, and at a moderate altitude, which were advantageous for potato production. Consequently, these areas not only produced high-quality and high-yield potato crops but also a high commodity rate and high input-output ratio.

The middle suitable areas were distributed throughout the 13 provinces in northern China. The average temperature during the potato growth season in this region was 15°C-18°C. It had sufficient light and heat in summer, large diurnal temperature variation, and few pests and diseases. Annual precipitation was approximately 200 mm-400 mm. The abundant precipitation was conducive to potato growth and development.

The generally suitable areas were mainly distributed in eastern Qinghai and eastern and southern Xinjiang. This area was located in the warm temperate zone, with adequate light and heat but scarce precipitation. The climate was hot in summer and cold in winter, with annual precipitation of approximately 200 mm. Temperatures fluctuated widely from day to day. The climate varied by region due to the complex topography. Large-scale potato cultivation was not seen in this region because of the numerous constraints to potato growth, though some climate conditions feasible for potato planting were found.

Areas with low suitability were mainly found in southern Qinghai. These areas were characterized by high altitude, cold climate, scarce rainfall and along frost season, rendering it unsuitable for potato growth.

### Effects of climate change on potato planting regionalization in northern China

**Fig 8:**
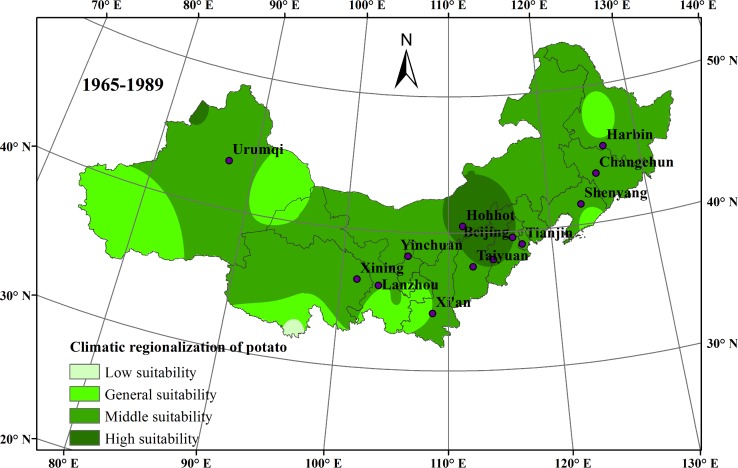
Climatic regionalization of potato planting in northern China during 1965–1989.

**Fig 9:**
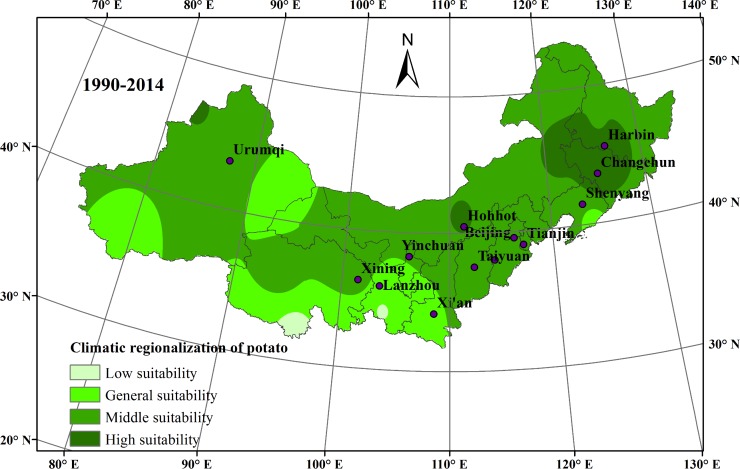
Climatic regionalization of potato planting in northern China during 1990–2014.

Variations in climatic regionalization of potato planting in northern China were compared during the first twenty-five years (1965–1989) in Fig 8 and the last twenty-five years (1990–2014) in Fig 9. The impact of climate change on different regions varied. For northeast China, climate change was very beneficial to local potato cultivation over the past 50 years, because of the expanded highly suitable areas for potato growth. For north China, the highly suitable areas receded and were replaced by moderately suitable areas. However, in northwest China, the potato planting was the most negatively affected by climate change. Especially, in the southern region of northwest China, the moderately suitable areas receded and were replaced by generally suitable areas. Meanwhile, in the western region of northwest China, the highly suitable areas receded and the low suitable areas expanded.

## Discussion

### Effects of climate change on potato planting suitability

It was generally found to be true that increasing temperature induced by climate warming tended to increase the developmental rate of crop and extend the length of growing season. In this study, we found the average temperature in northern China increased significantly from 17.52 in 1965 to 18.51 in 2014. This would result in a positive impact on potato production at higher latitude seven in the absence of improvements in cultivars, cultivation, and management [20]. However, when current temperatures approach the upper limits of the optimal temperature range for potato growth, higher temperatures will not only detrimentally affect vine and root growth but also might cause a delay in tuber initiation and consequently a reduction in final potato yield [31,32]. In addition, tuber yield and the depth and density of the potato rooting system might be considerably reduced in response to drought [33,34]. In this study, we found the annual precipitation in northern China decreased during the period of 1965–2014 and fluctuated greatly in the interannual range. Decreased precipitation in many areas was predicted to increase the need for irrigation of potato crops [35], especially while tubers were growing. In general, potatoes are long-day plants, strongly relying on light which affects not only growth but also assimilation and distribution. In this study, we found the sunshine hours in northern China from 1965 to 2014 decreased from 8.67 hours in 1965 to 7.33 hours in 2014, which might be related to the decreasing sunny days[36].

The climatic suitability index accounted for the effects of light, temperature and precipitation on the growth and development of the potato. As the climate changed, zones suitable for potato cultivation shifted. For farmers and advisors, it was critically important to understand these changes in order to develop short-term and long-term adaptation strategies with limited resources [20,37]. In this study, we found that the middle suitability cropping areas and high suitability cropping areas for potato over the past 50 years receded in northwest China and north China, respectively. Furthermore, the high suitability cropping areas expanded in northeast China, mainly because potatoes belonged to the cool season crops. However, increased heat and reduced water resources caused by climate warming in northwest and north China had negative effects on the distribution of areas suitable for potato planting. These results were consistent with a number of previous studies [18,21,38]. For example, Wang et al [18] found that the areas suitable for the potato in northwest China would move northward and recede as a result of climate change in the future. Zhao et al [21] pointed out that the key meteorological factors affecting potato yield in northern China was diurnal temperature range, radiation, precipitation and reference crop evapotranspiration based on daily climate variables obtained from 607 meteorological stations from 1961 to 2014 and detailed field experimental data, which would have an impact on the planting distribution of potato. Tang et al [38] indicated that variation in potato yield in North China correlated significantly with variation in growing-season effective precipitation, as well as variation in the ratio of precipitation to potential evapotranspiration during the tuberization stage, but not with variation in growing-season total precipitation. Thus, the impact of climate warming and drying in North China had negative effects on the distribution of areas suitable for potato planting.

### Limitations and implications

This study focused on exploring the variations in climatic suitability and planting regionalization for potato in northern China under climate change. The results provided a scientific basis for sustainable potato production in northern China to adapt to future climate change. However, the impacts of climate change on potato suitability and planting regionalization in northern China are complex, with many attendant uncertainties. Several areas of uncertainty identified in this study may outweigh the final results. First, because of collected data limitations, potato growth season in this study wasonly divided into 3 stages (from sowing to emergence, from emergence to flowering and from flowering to maturity), and more detailed stages of development were not fully taken into account, possibly obscuring the results. It is generally believed that the potato growth season can be divided into 6 stages: germination period (bud sprout from stem to emergence), seedling stage (emergence to bud), tuber formation period (bud to beginning flower), tuber growth period (flower to senility of stem and leaf), starch accumulation period (senescence to senility of stem and leaf) and dormancy period of the tuber (fully mature or harvested). It is noteworthy that potential differences among the growth stages were found in response to differences in the local climate and type of management system. Second, the comprehensive production effect on potato planting was ignored in this study and was therefore a source of uncertainty. In this study, the suitability of potato planting in northern China was investigated from the perspective of the development and utilization of climate resources. In practice, potato production and industrial development must be comprehensively considered in terms of climate conditions, industrial policy, market supply and demand, agricultural technology advancement, and so on. Third, as for the comprehensive climate suitability of the potato, the weight was determined by the correlation coefficient method in this study. There are many ways to determine the weight, such as the empirical method and the multi-actor statistical method [7,21]. It is well-known that each method has its advantages and disadvantages. Therefore, there were differences in the final results caused by the calculation methods.

Despite these shortcomings, these results do offer a more comprehensive understanding of the impact of climate change on potato suitability and planting regionalization in northern China at the regional scale. The variations in climatic suitability and planting regionalization for potato in northern China over the past 50 years have been quantitatively evaluated in our study. In order to improve farming management and invest in more facilities in practice, a subsequent study is necessary to explore the distribution pattern of climatic suitability for the potato in future climate scenarios in northern China.

## Conclusions

The results presented in this paper demonstrated the variations in climatic suitability and planting regionalization for the potato in northern China from 1965 to 2014. Over the past 50 years, the comprehensive climatic suitability index during the potato growth season in the study area fluctuated but showed a slight increase overall. This increase would be beneficial to potato production in the vast area of northern China. Specifically, the temperature suitability index and the precipitation suitability index both decreased with at rates of 0.002 and 0.008 every ten years, respectively, and the reduction in the precipitation suitability index (0.006) was greater than that in the temperature suitability index (0.018). However, the light suitability index and comprehensive climatic suitability index exhibited increasing trends, at rates of 0.014 and 0.002 every ten years, respectively. Moreover, the increase in light suitability index (0.035) was greater than that in comprehensive climatic suitability index(0.004) over the past 50 years. For the spatial distribution, the average suitability indexes of temperature, precipitation and comprehensive climate all showed gradually declining trends from the eastern region to the western region, expanding westward and southward from 1965 to 2014. For the growth period, the comprehensive climatic suitability index increased during the sowing-emergence stage. However, it decreased during the emergence-flowering stage and the flowering-maturity stage, which would directly affect the quality and yield of potato, particularly because the flowering-maturity stage was associated with potato tuber enlargement and starch accumulation. The effects of climate change on potato planting regionalization in northern China over the past 50 years were evident. It was particularly noted that climate change was more beneficial to potato cultivation in northeast China, where highly suitable areas had obviously expanded. However, the planting of potatoes was the most negatively affected in northwest China, where middle suitable areas had receded.

In summary, these results have important implications for policy-makers attempting to develop effective and sustainable strategies for agricultural development to mitigate the effects of climate change. For a more complete assessment of agricultural climatic resource suitability and potato production in this area, future work will need to consider additional factors such as extreme weather and crop varieties through field-based studies.

## Supporting information

S1 File (ZIP)
