# Supplementary material for: Variations in climatic suitability and planting regionalization for potato in northern China under climate change
Source: PLoS One. 2018 Sep 27;13(9):e0203538. doi: 10.1371/journal.pone.0203538 (PMC6159864; doi:10.1371/journal.pone.0203538)
Supplement: S1 File — (ZIP) [file pone.0203538.s001.zip › S1_File/Table_1.docx]

**Table 1.** Temperature parameters of potato during thedifferent growth stages in northern China.

| Variable | Growth stage | | |
| --- | --- | --- | --- |
|  | From sowing to emergence | From emergence to flowering | From floweringto maturity |
| T_1_ | 5 | 7 | 8 |
| T_0_ | 16 | 19 | 17 |
| T_2_ | 25 | 30 | 29 |
| B | 0.82 | 0.92 | 1.33 |

Notes:*T_1_*_,_*T_0_* and *T_2_*were the lower limit of growth temperature, the most suitable growth temperature and the upper limit of growth temperature, respectively; B was constant.
