# Supplementary material for: Variations in climatic suitability and planting regionalization for potato in northern China under climate change
Source: PLoS One. 2018 Sep 27;13(9):e0203538. doi: 10.1371/journal.pone.0203538 (PMC6159864; doi:10.1371/journal.pone.0203538)
Supplement: S1 File — (ZIP) [file pone.0203538.s001.zip › S1_File/Table_2.docx]

**Table 2.** Crop coefficient of potato during thedifferent growth stages in northern China.

| Variable | Growth stage | | |
| --- | --- | --- | --- |
|  | From sowing to emergence | From emergence to flowering | From floweringto maturity |
| Kc | 0.4 | 1.15 | 0.75 |
