# Supplementary material for: Variations in climatic suitability and planting regionalization for potato in northern China under climate change
Source: PLoS One. 2018 Sep 27;13(9):e0203538. doi: 10.1371/journal.pone.0203538 (PMC6159864; doi:10.1371/journal.pone.0203538)
Supplement: S1 File — (ZIP) [file pone.0203538.s001.zip › S1_File/Table_3.docx]

**Table 3.** Key climate factors weights during the potato growing seasonin northern China.

| Weight value | Major climatic factors | | |
| --- | --- | --- | --- |
|  | Temperature | Precipitation | Light |
| w | 0.37 | 0.30 | 0.33 |
