# Supplementary material for: Variations in climatic suitability and planting regionalization for potato in northern China under climate change
Source: PLoS One. 2018 Sep 27;13(9):e0203538. doi: 10.1371/journal.pone.0203538 (PMC6159864; doi:10.1371/journal.pone.0203538)
Supplement: S1 File — (ZIP) [file pone.0203538.s001.zip › S1_File/Table_4.docx]

**Table 4.** Climatic regionalization of potato planting in northern China.

| C value | Comprehensive climate suitability | Climatic regionalization level for potato planting |
| --- | --- | --- |
| 0.80-1.00 | High suitability | Ⅳ |
| 0.65-0.80 | Middle suitability | Ⅲ |
| 0.55 -0.65 | General suitability | Ⅱ |
| 0.00 - 0.55 | Low suitability | Ⅰ |
