# Supplementary figures and images for: Variations in climatic suitability and planting regionalization for potato in northern China under climate change
Source: PLoS One. 2018 Sep 27;13(9):e0203538. doi: 10.1371/journal.pone.0203538 (PMC6159864; doi:10.1371/journal.pone.0203538)

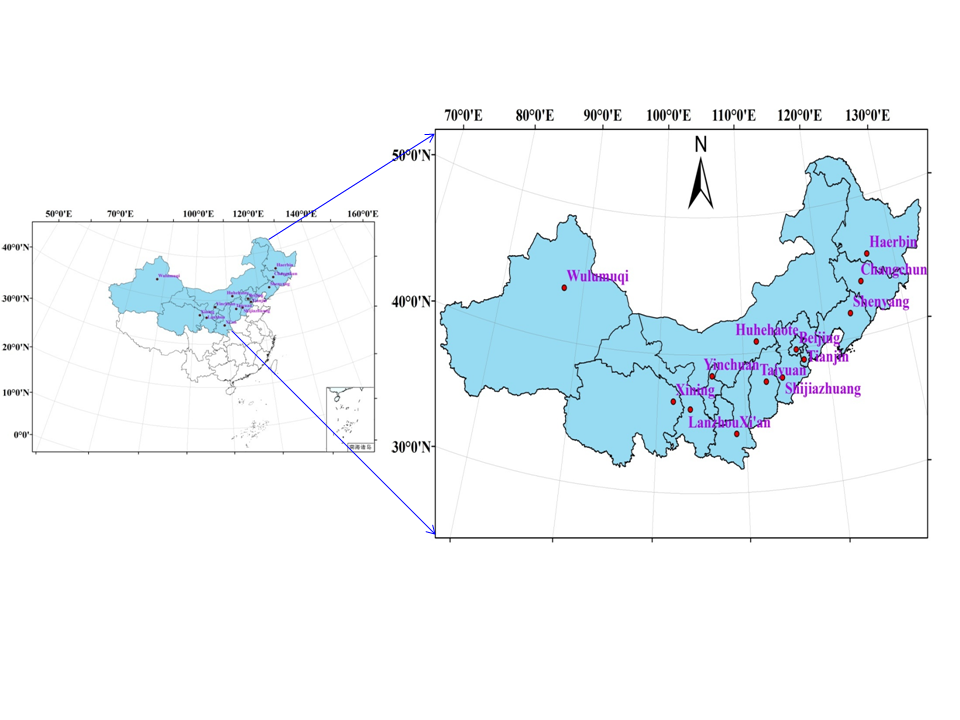

Supplement: S1 File — (ZIP) [file pone.0203538.s001.zip › S1_File/Fig_1.tif]

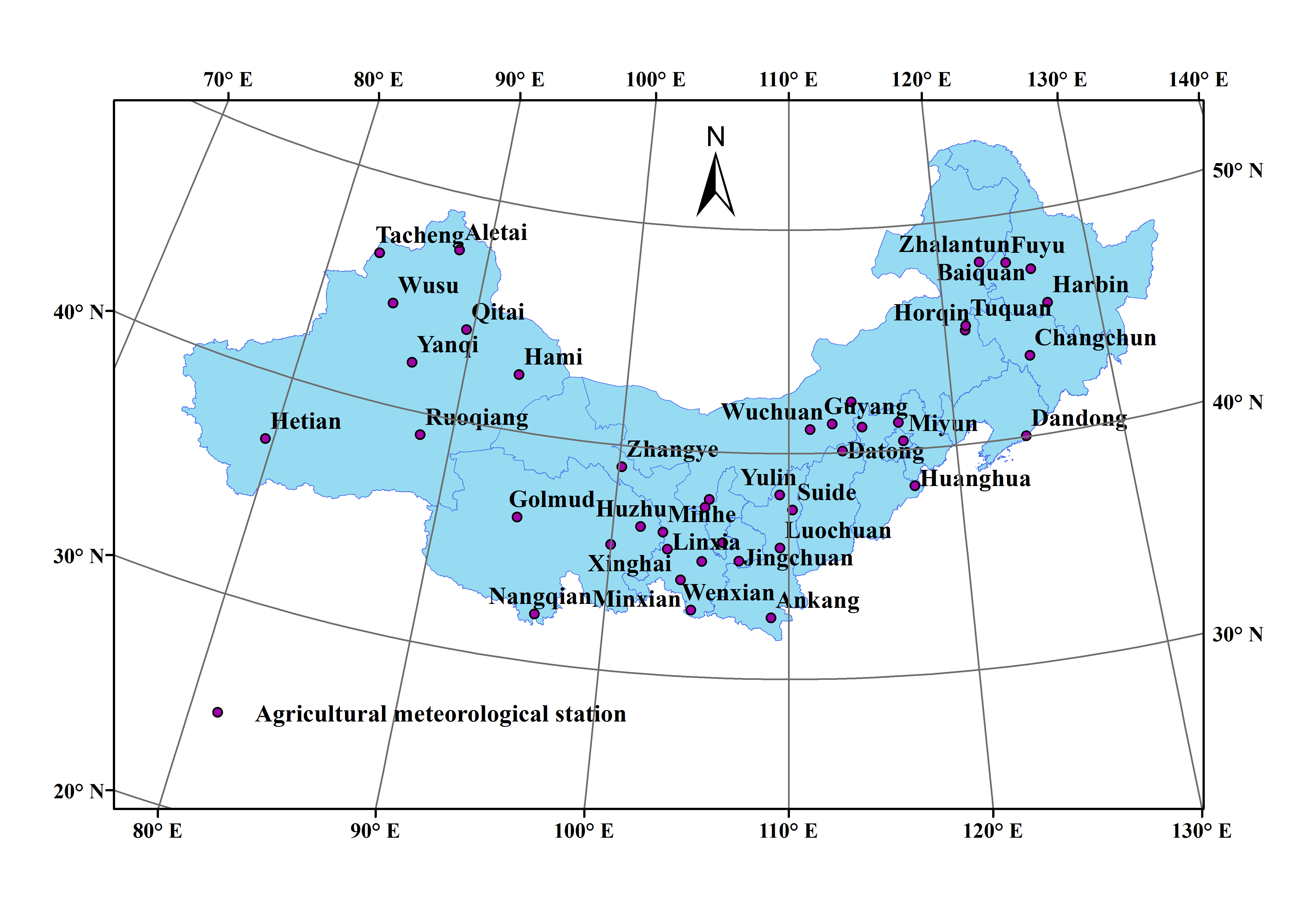

Supplement: S1 File — (ZIP) [file pone.0203538.s001.zip › S1_File/Fig_2.tif]

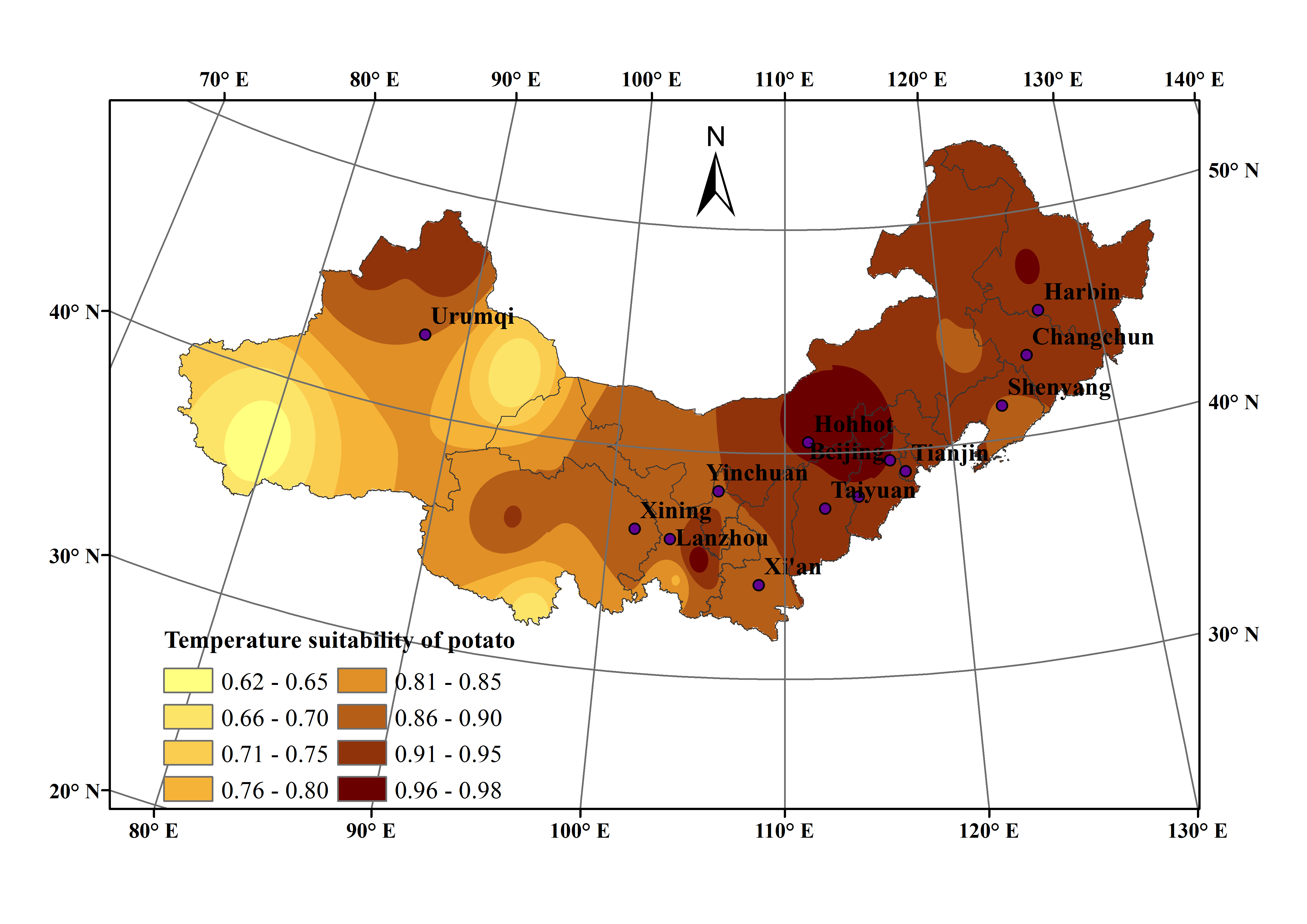

Supplement: S1 File — (ZIP) [file pone.0203538.s001.zip › S1_File/Fig_3.tif]

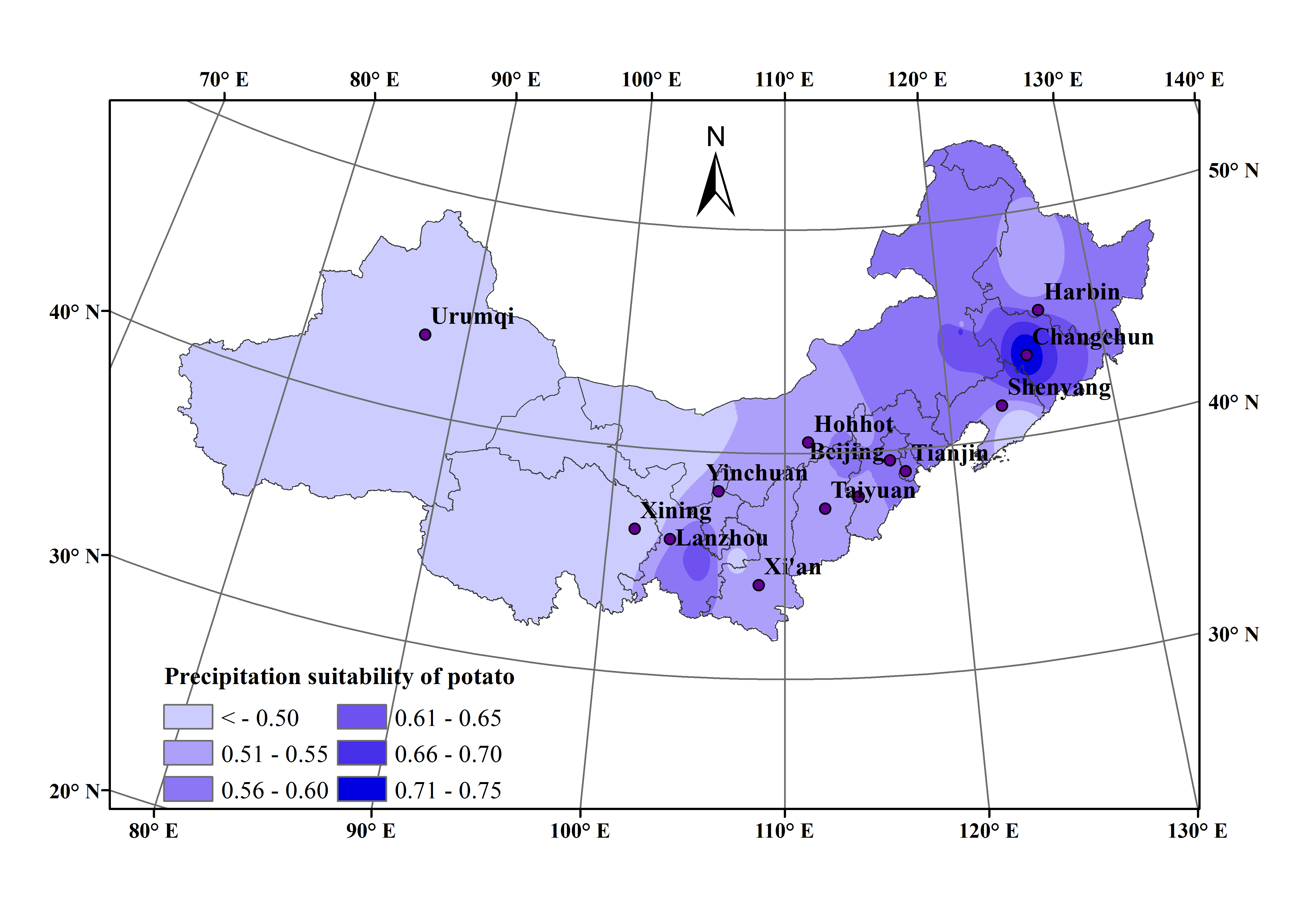

Supplement: S1 File — (ZIP) [file pone.0203538.s001.zip › S1_File/Fig_4.tif]

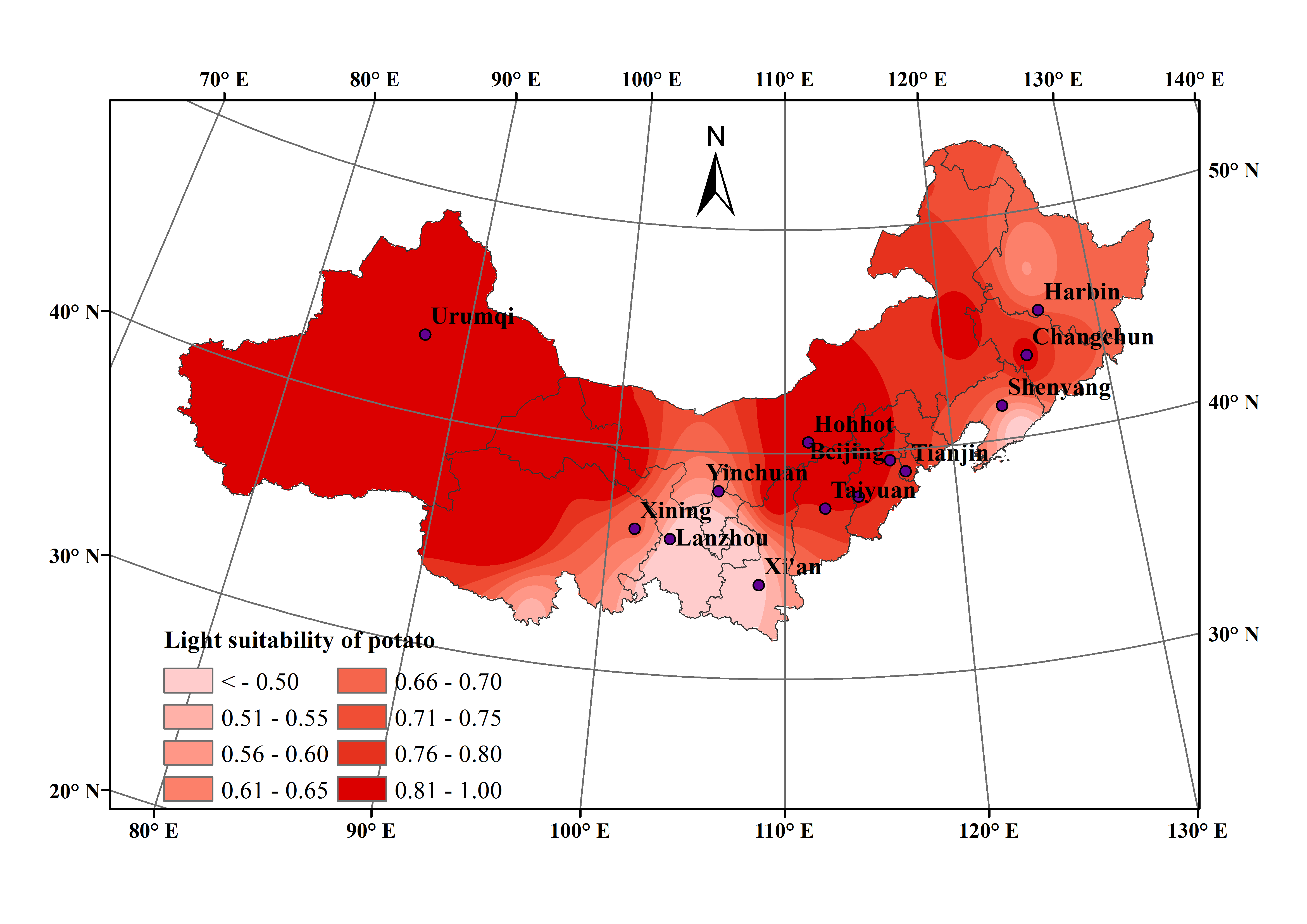

Supplement: S1 File — (ZIP) [file pone.0203538.s001.zip › S1_File/Fig_5.tif]

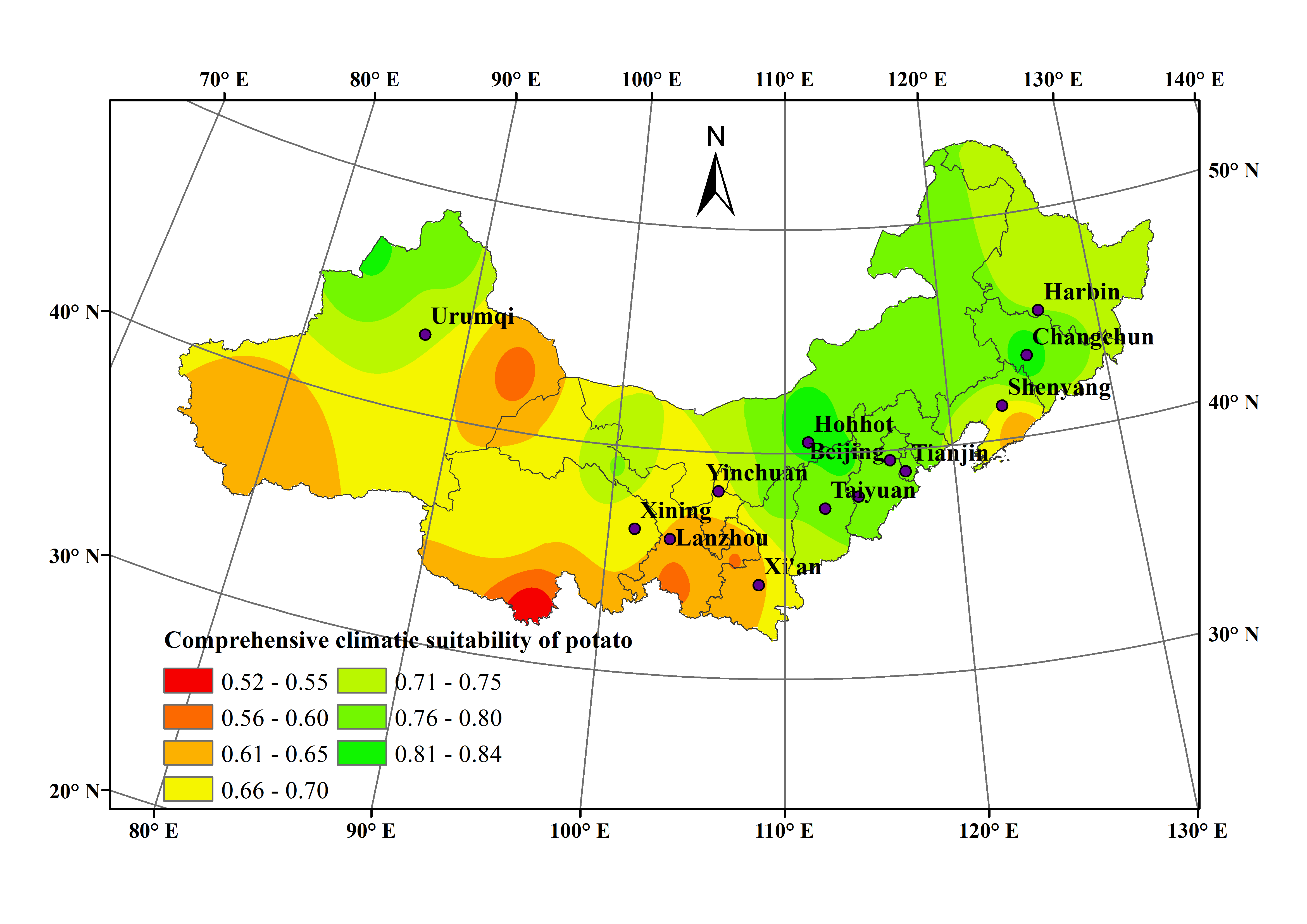

Supplement: S1 File — (ZIP) [file pone.0203538.s001.zip › S1_File/Fig_6.tif]

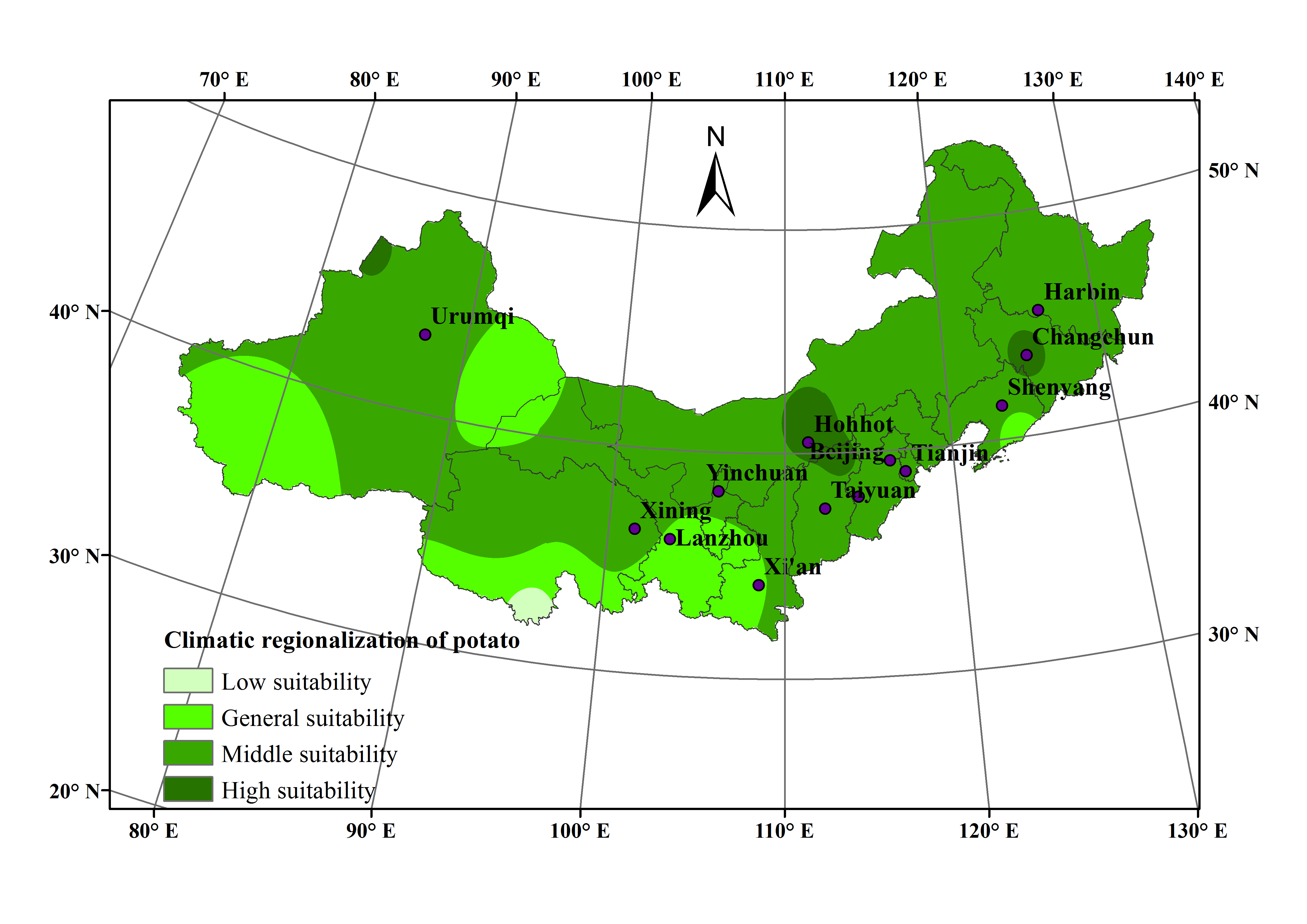

Supplement: S1 File — (ZIP) [file pone.0203538.s001.zip › S1_File/Fig_7.tif]

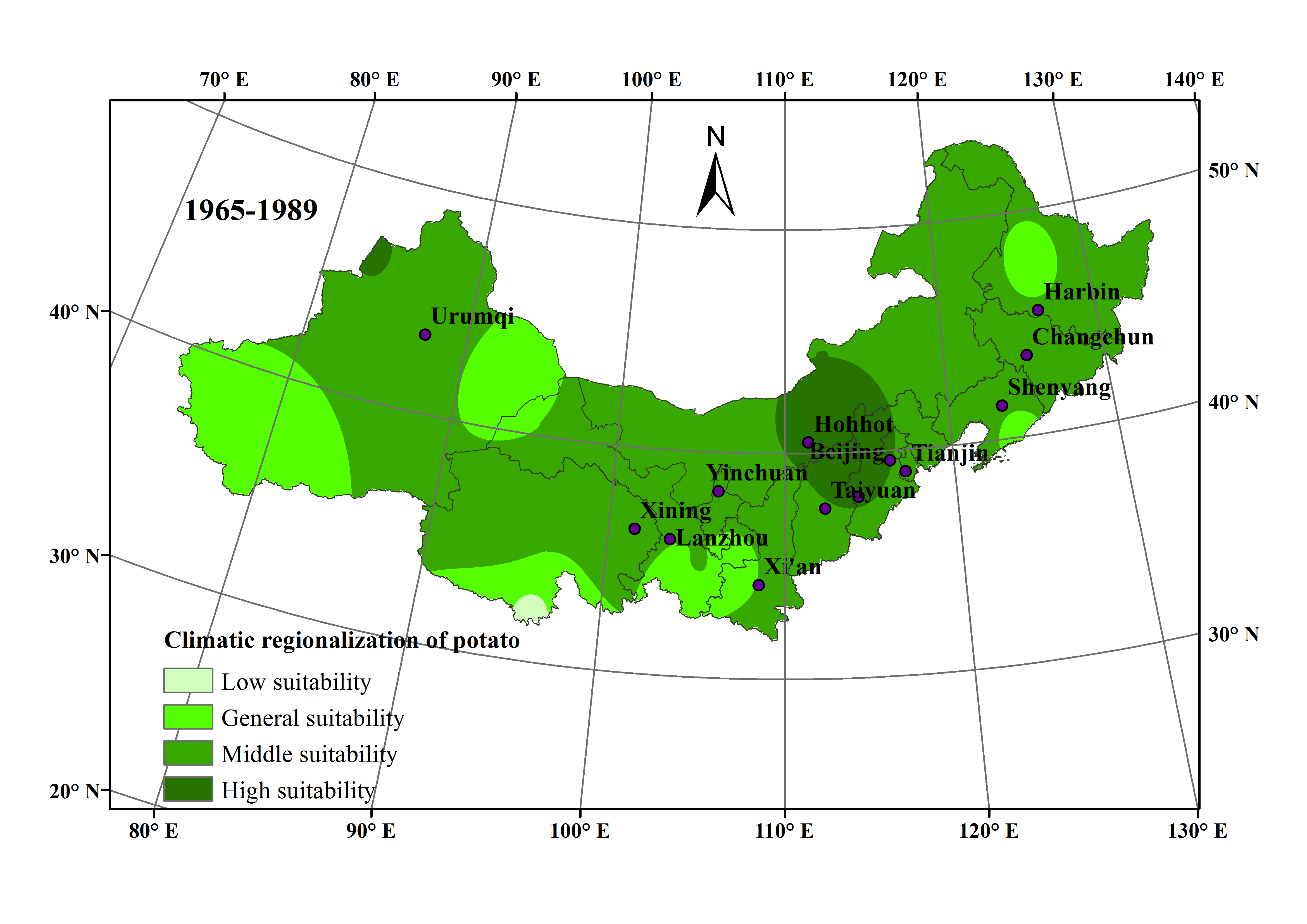

Supplement: S1 File — (ZIP) [file pone.0203538.s001.zip › S1_File/Fig_8.tif]

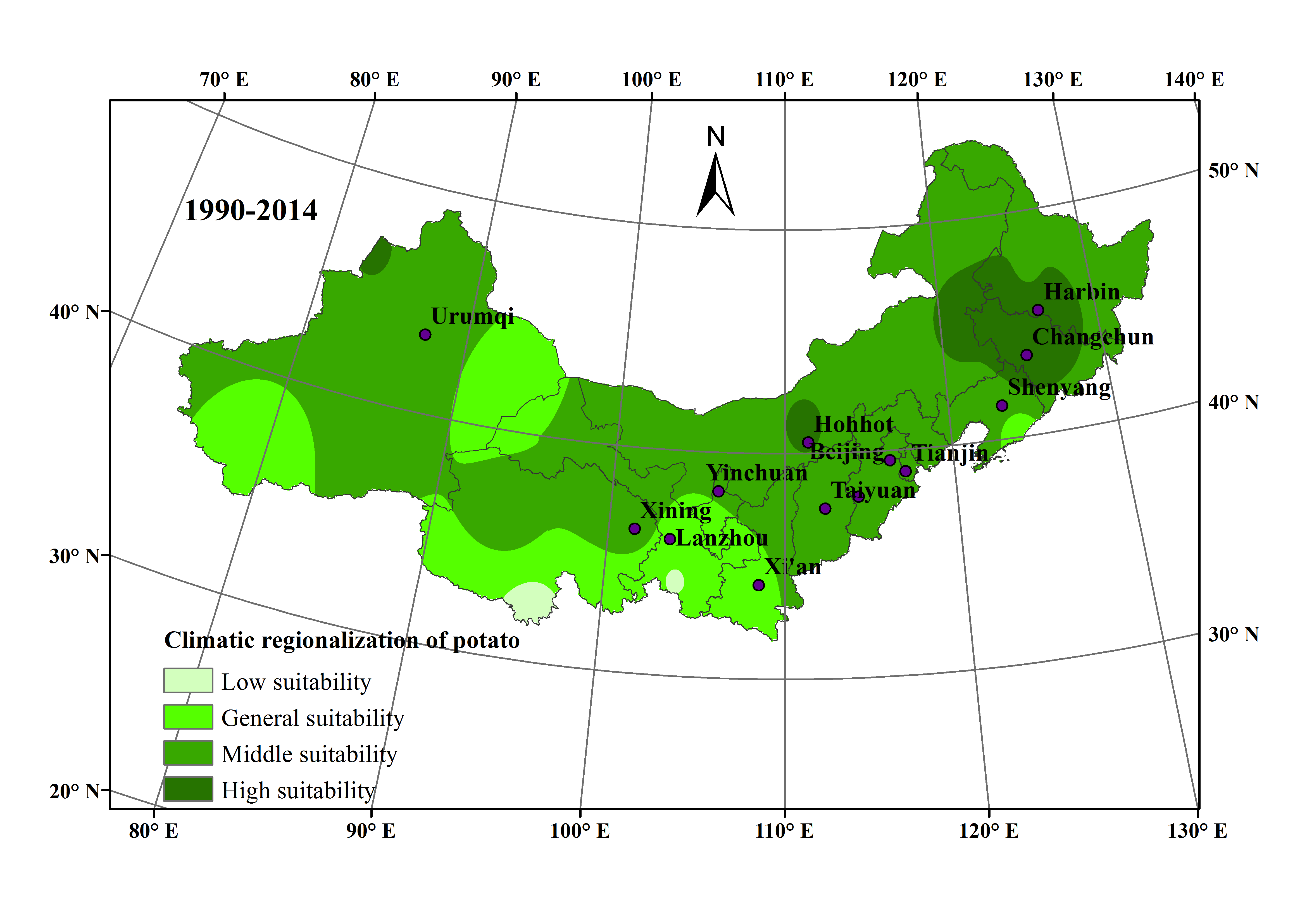

Supplement: S1 File — (ZIP) [file pone.0203538.s001.zip › S1_File/Fig_9.tif]
